# Nurturing maternal health in the midst of difficult life circumstances: a qualitative study of women and providers connected to a community-based perinatal program

**DOI:** 10.1186/s12884-018-1951-6

**Published:** 2018-08-03

**Authors:** Maira Quintanilha, Maria J. Mayan, Kim D. Raine, Rhonda C. Bell

**Affiliations:** 1 Agricultural, Food & Nutritional Science, University of Alberta, 4-126 Li Ka Shing Centre for Health Research Innovation, Edmonton, AB T6G 2E1 Canada; 2 Faculty of Extension, University of Alberta, Community-University Partnership, 2-281 Enterprise Square, 10230 Jasper Avenue, Edmonton, AB T5J 4P6 Canada; 3 School of Public Health, University of Alberta, 4-077 Edmonton Clinic Health Academy, Edmonton, AB T6G 1C9 Canada

**Keywords:** Pregnancy, Postpartum, Rural women, Community-based programs, Qualitative, Focused ethnography, Canada

## Abstract

**Background:**

Many socioecological and structural factors affect women’s diets, physical activity, and her access and receptivity to perinatal care. We sought to explore women’s and providers’ perceptions and experiences of health in the pre- and post-natal period while facing difficult life circumstances, and accessing a community-based program partially funded by Canada Prenatal Nutrition Program (CPNP) in Alberta, Canada.

**Methods:**

Following the principles of community-based participatory research, we conducted a focused ethnography that involved five focus groups with women (28 in total), eight one-on-one interviews with program providers, and observations of program activities. Data were analyzed through qualitative content analysis to inductively derive codes and categories.

**Results:**

Women perceived eating healthy foods, taking prenatal vitamins, and being physically active as key health behaviours during pregnancy and postpartum. However, they were commonly coping with many difficult life circumstances, and faced health barriers for themselves and their babies. These barriers included pregnancy or birth complications, family and spousal issues, financial difficulties, and living rurally. On the other hand, women and providers identified many aspects of the community-based program that addressed the burden of adversities as enablers to better health during pregnancy and postpartum.

**Conclusion:**

Community-based programs have an important role in alleviating some of the burden of coping with difficult life circumstances for women. With such potential, community-based programs need to be well supported through policies. Policies supporting these programs, and ensuring adequate funding, can enable more equitable services to rural women and truly promote maternal health during pregnancy and postpartum.

**Electronic supplementary material:**

The online version of this article (10.1186/s12884-018-1951-6) contains supplementary material, which is available to authorized users.

## Background

Women’s diets, physical activity, along with their access and receptivity to perinatal care can significantly impact maternal health and pregnancy outcomes [1, 2]. However, many socioecological factors affect health behaviours during pregnancy and postpartum [3–5]. Research with low-income, pregnant women suggests that they perceive multiple life “hardships” (e.g., custody issues, child care, lack of social support, etc.) as factors that increase their stress and decrease their overall self-efficacy for healthy behaviours in relation to diet and physical activity throughout the prenatal period [3]. In this study, we refer to life hardships that negatively affect women’s experiences as “difficult life circumstances.”

Food insecurity – defined as “inadequate or insecure access to food because of financial constraints” [6] – commonly occurs in difficult life circumstances and is associated with nutrient deficiencies, depressive symptoms, and a higher sense of stress for mothers, as well as poor birth outcomes for infants [7–9]. As a stress-relief mechanism or as a strategy to eat in low-cost ways, pregnant women who experience household food insecurity might consume more foods that are high in energy, fat, and refined carbohydrates (e.g., sugar) [3, 9]. Food insecurity during pregnancy has been associated with poor dietary intake with decreased consumption of vegetables and fruit, and lower micronutrient intake [9, 10].

In Canada, women who are coping with difficult life circumstances (low income, teen pregnancy, social and geographic isolation, substance use, family violence, and recent immigration) [11] and become pregnant can access programs offered through the Canada Prenatal Nutrition Program (CPNP) during pregnancy and up to 6 months postpartum. CPNP programs are supported by the Public Health Agency of Canada (PHAC) in conjunction with provincial/territorial governments and community-based organizations as appropriate, and focus on maternal/child nutrition and health of pregnant and postpartum women facing difficult life circumstances. Each CPNP program delivery is unique but follows six guiding principles: mothers and babies first, equity and accessibility, community-based, strengthening and supporting families, partnerships, and flexibility to appropriately respond to women’s different needs in each community [12]. Currently, there are approximately 276 CPNP programs providing support to ~ 51,000 women across Canada, with 21 programs in rural and urban areas of Alberta [13].

This study is part of a larger research program called ENRICH. The ENRICH Research Program began in 2013 with the overall purpose of promoting maternal health in pregnancy and postpartum, among diverse groups of women in Alberta, through healthy eating [14]. In the study presented here, we sought to explore how difficult life circumstances shaped pregnant and postpartum women’s perceptions and experiences of health. We were particularly interested in understanding how difficult life circumstances were intertwined, and were perhaps intensified because of pregnancy, postpartum, and “rurality” (women’s residence in rural Alberta) [15].

## Methods

We followed the principles of community-based participatory research (CBPR) to engage pregnant and postpartum women, as well as health care and service providers who were connected with them through a CPNP program. The CBPR approach [16] enabled researchers to develop relationships with community-based health care and service providers and involve them in identifying questions for women and appropriate methods for data generation, and in the interpretation of data.

Within the principles of CBPR, we used a focused ethnography methodology as it is sensitive to how culture shapes, and possibly explains, our everyday lives and health behaviours [17, 18]. It is also appropriate for investigating strategies to improve health delivery systems provided it links everyday health care issues, and interactions with health care providers, with wider cultural norms “with emphasis on context” [18]. In contrast to traditional ethnography, focused ethnography is more contained to a certain setting, concentrated on an issue or on a shared experience, and completed within a shorter time frame. “Culture” was defined as the shared experience of pregnancy and postpartum among women living with difficult life circumstances, and accessing a community-based program in rural Alberta.

### Setting

We conducted the study with pregnant and postpartum women connected through the CPNP *Healthy Moms Healthy Babies* (HMHB) program (with in-kind support from Alberta Health Services) across five rural communities in Southern Alberta. Although some participants lived in bigger rural communities that were geographically close to large metropolitan areas, women described how living rurally might have shaped their experiences in pregnancy and postpartum; thus, “rurality” in our research was socially constructed by women [14].

The CPNP funding allocated to the HMHB program was used for three part-time positions and for program activities. The HMHB program setting was purposefully selected because it allowed us to work with women during pregnancy and up to 6 months postpartum, and facing at least two difficult life circumstances (low income, teen pregnancy, social and geographic isolation, substance use, family violence, recent immigration), which were CPNP/HMHB intake criteria [11].

### Recruitment & sampling

In order to recruit HMHB providers, we attended two of their monthly meetings to discuss the aims of the research. Those who expressed interest in participating were asked to contact one of the researchers via e-mail or phone. We used purposeful sampling to identify providers who delivered HMHB services, had a good understanding of the program and clientele, and consistently met with women during pregnancy and postpartum.

One of the main HMHB program activities was *cooking circles* where providers organized a time when women cooked a meal together at a minimal cost of one dollar per serving, while having the opportunity to socialize. In a cooking circle a month prior to researchers’ scheduled visit, HMHB providers explained the overall purpose of the study, and gauged women’s interest in participating. Convenience sampling was used in that all rural women who were HMHB clients could discuss their health perceptions and experiences during pregnancy and postpartum while facing difficult life circumstances [19]. Given the CBPR approach taken in the project, all HMHB clients who wanted to participate were included. Women and providers provided signed informed consent. The Research Ethics Board at the University of Alberta approved all aspects of the research.

### Data generation

We conducted five focus groups (FGs) with women (approximately six per group, total of 28 women) across five diverse Southern Alberta rural communities. Of these women; 25 were postpartum and three were pregnant, four were immigrants (three from Southeast Asia and one from South America), and they had an average of two children. FG were conducted by one moderator (MQ), with the assistance of another researcher who took note of facial expressions and occasional side conversations. HMHB providers discussed the research with women a week prior to FGs, and because all women who regularly attended cooking circles were interested in participating, HMHB providers indicated to researchers that it would be best for FGs to take place before scheduled cooking circles. However, HMHB providers were not present during FGs so that women could feel more comfortable in discussing their experiences with the program.

FGs were an appropriate method of data generation as women who shared similar life circumstances were provided with a nonthreatening, nonjudgmental setting to discuss a range of health topics [20]. The FG moderator used a focus group guide to ask open-ended questions and probe women about their health perceptions, experiences in relation to health, challenges, and supports during pregnancy and postpartum (Additional file 1). We conducted FG in all rural communities where HMHB providers held cooking circles for women. In addition, we conducted eight semi-structured one-on-one interviews with HMHB providers (e.g., public health nurses, dietitians, food coordinators, outreach workers) who worked in each of the five communities. For interviews, we used a topic guide with exploratory questions about how HMHB providers supported women (i.e., HMHB clients), and their organizational contexts. We conducted interviews until data saturation [21] was reached. Both FGs with women and interviews with providers were audio-recorded.

We also actively engaged with women and HMHB service providers during cooking circles, and took this opportunity for data generation through participant observation, adopting the “observer-as-participant” role [18]. We accumulated approximately 12 h of participant observation in cooking circles across the communities. These observations enabled us to learn more about the interactions between women and HMHB providers, and comments women commonly shared with them, and how providers responded to women’s needs and/or concerns. Participant observations were captured through researchers’ audio-recorded debriefings after each cooking circle. These debriefings included descriptive information of cooking circle settings and activities in addition to researchers’ initial reflections on data generated at each rural community visit. Researchers also kept notes on focus groups to provide additional context, and recorded analytic comments on participation, women’s interactions, and facial expressions.

### Data analysis

Audio recordings of focus groups, interviews and debriefings were transcribed verbatim. Data were managed using NVivo (Version 11, QSR International), and analyzed using qualitative content analysis to inductively derive categories [21, 22]. One researcher (MQ) was responsible for coding transcripts, and bringing emerging categories to all involved researchers for review, discussion, and verification. Later on, MQ brought emergent categories to HMHB providers for further discussion and more in-depth interpretation. Due to the large amount of data generated in a short period of time, we followed an inductive, cyclic, iterative, self-reflective analytic process [18]. HMHB clients’ data were used to build the primary story. Data collected through providers’ interviews and observations were key in enriching women’s description and helping us understand the role of a CPNP/community-based program in their pregnancy and postpartum experiences.

## Results

**Fig. 1:**
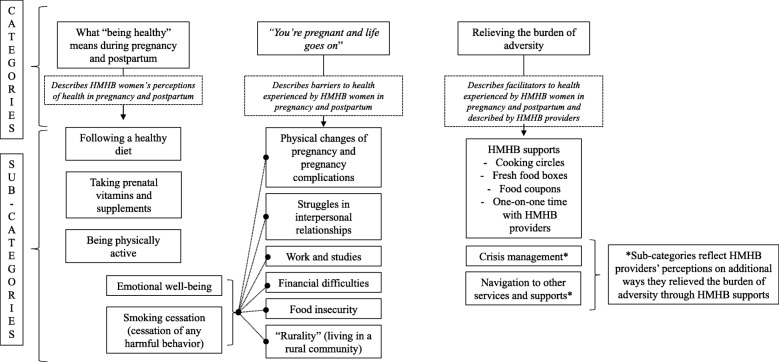
Categories and sub-categories that emerged from the data

**Fig. 2:**
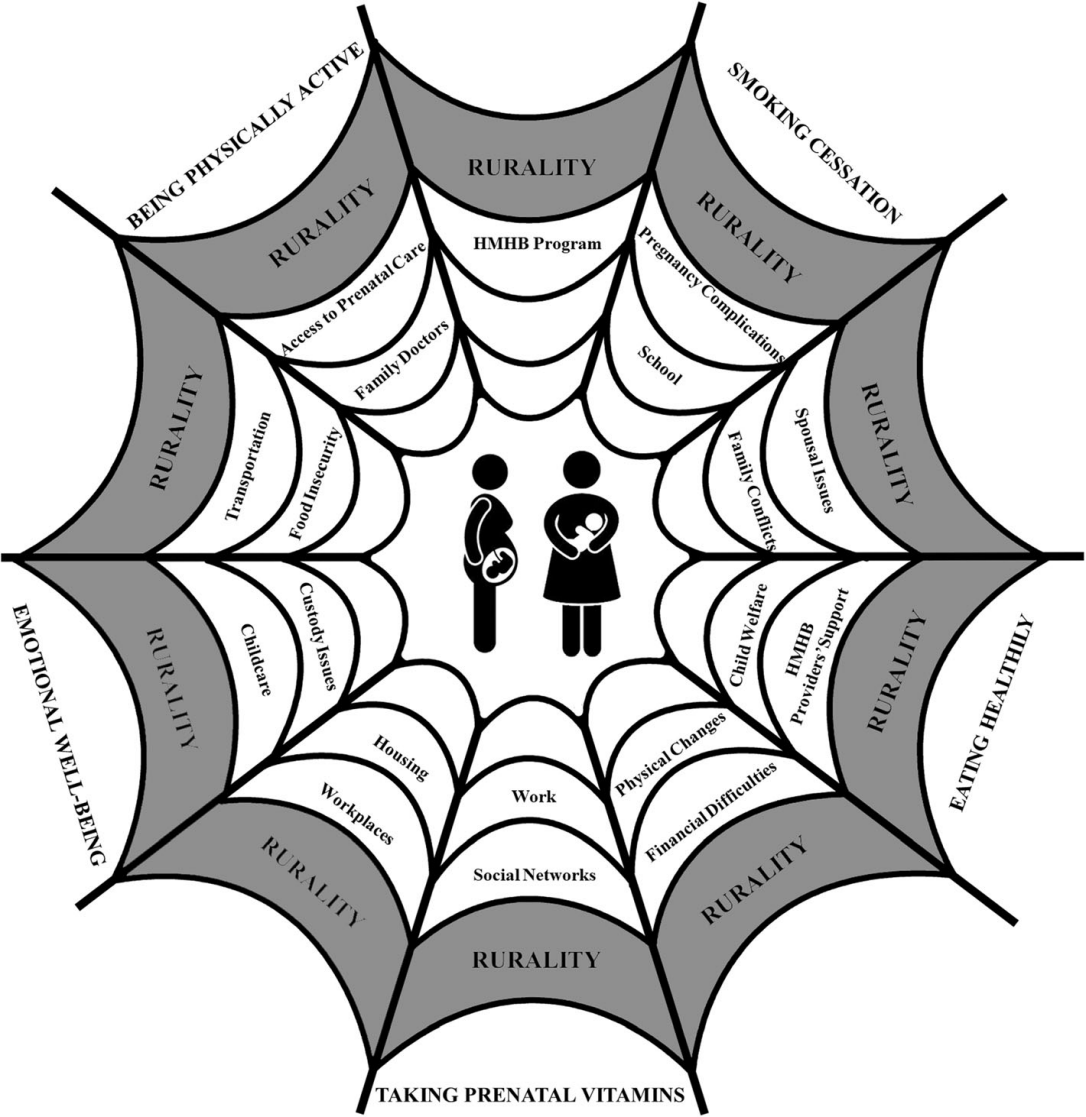
The web of factors shaping women’s experiences in pregnancy and postpartum

Our results provide a rich description of what “being healthy” during pregnancy and postpartum meant for women accessing a CPNP program, and how difficult life circumstances and participation in the program shaped women’s health experiences. Categories and sub-categories that emerged from our data are presented in Fig. 1, and described in more detail here. Additionally, we present them in a way that shows how they can interconnect, forming a web of factors (Fig. 2).

### What “being healthy” meant to HMHB clients during pregnancy and postpartum

Women from diverse rural communities perceived their babies’ health as an extension of their own health during pregnancy, as well as postpartum. When we asked women what it meant for them to be healthy during pregnancy and postpartum, they highlighted the importance of following a healthy diet, taking prenatal vitamins and/or any supplements prescribed by their doctors, being physically active, looking after their emotional well-being, and stopping any behaviour they perceived as harmful to their babies (i.e., smoking):

*“It’s to eat so that the baby gets all the nutrients it needs to grow, like, lots of fruit, vegetables. That’s what healthy meant to me. I always thought, whatever I eat, she eats so what would I feed a small child? So that’s basically what I ate. I kept healthy. I was really bad before I was pregnant, like, I never cooked or anything so, yeah, I changed.” (Postpartum woman, mother of two)*

*“It’s number one to try and be as stable as possible for my son because he picks up everything and with my daughter being so young, she’s a tiny little sponge. Like, she doesn’t understand what we’re saying or anything but she can grasp the moods in the room. If mommy’s tense, she starts crying so it’s very - I have to be very calm for both of them, which, for me, is very hard because I’m not that type of person to begin with.” (Postpartum woman, mother of two)*

Description of a healthy diet varied among women and between groups. We did not probe women in each FG to provide us with a detailed definition of what a healthy diet meant for them, yet some commonly described the following: eating more fruit and vegetables; increasing intake of iron-rich foods, such as meat and dark leafy greens; increasing intake of milk and alternatives; and managing sweet cravings and sugar consumption. Despite individual struggles related to diet, women provided various examples in which they attempted to have a healthy meal or diet by balancing nutritious foods (e.g., broccoli, carrots, fish) with less nutritious foods that had a high sugar and fat content (e.g., burger, ice cream, milkshake, potato chips):

*“Well if you had a craving for like a cheeseburger, you eat the cheeseburger, but then later you eat like a handful of carrots or broccoli or something just to balance it out because, I don’t know, I thought, you’re not getting a whole lot of vitamins out of that cheeseburger.” (Postpartum woman, mother of two)*

Prenatal vitamins and supplements were another common nutrition-related topic women brought up when discussing their perception of health during pregnancy. They talked about brands, where to buy them, the size of the pills, and symptoms they might have experienced because of their daily intake. Even though some women described having difficulty in swallowing prenatal vitamins or simply disliking taking them, they still took them when they were pregnant because it was something they commonly felt they had to do out of respect for their health care providers’ advice: *“I never take medication, like ever, and then when I’m pregnant, I have to suck it up and do it” (Postpartum woman, mother of three)*.

Being physically active was also described as a key element of being healthy during pregnancy. Women discussed how “exercising” had to be adequate for pregnancy – *“not to go overboard”* but not to *“sit on the couch all day”* – and fit into their already busy routines. Women across communities seemed unsure about adequate physical activity during pregnancy and only did what made them feel comfortable. This commonly translated into walking and maintaining their usual household and non-sedentary work activities, including cleaning, standing for long stretches of time during their work day, and looking after older children. Women’s perceptions of being physically active shifted after having a baby, as they commonly described exercise as a strategy to cope with postpartum stress and to reduce isolation.

#### *“You’re pregnant and life goes on”*

This section describes barriers to women’s health during pregnancy and postpartum, and how these commonly affected women’s emotional well-being and increased the stress in their lives. We also show how barriers turned into entry points for HMHB providers to promote their clients’ maternal health amid many social and health adversities.

Physical changes of pregnancy and the onset of pregnancy or birth complications were described as barriers to being healthy during pregnancy and postpartum. The tiredness, nausea, vomiting, and mood changes commonly experienced during pregnancy hindered women’s ability to eat healthily and be physically active. It also added more stress to women’s lives and further complexity to whatever situation they were already experiencing. Although becoming a mother was very important to the women, there were personal and professional factors in their lives’ “equation” that made the physical and emotional changes of pregnancy and postpartum harder to handle.

This was particularly discussed among those who were struggling in the relationships with their boyfriend, partner, or spouse. Some women were in situations where partner/spousal abuse existed, and working through difficulties in their relationships while pregnant meant leaving the father of the baby, finding housing, fighting for custody, and ensuring their children’s safety. In the following quote, a mother of two, who left an abusive partner and fought for custody during her second pregnancy, described how her experience added stress to a time in her life when she was already feeling emotional:

*“Your hormones take over quite a bit because you get so emotional. So, I thought that was the hardest part just because of what my family life was going through and dealing with all of that at the same time was stressful. So, if things were settled and calm, I’m sure it would have been easier to deal with, but for me it was pretty hard.” (Postpartum woman, mother of two)*

In addition to what was happening in their personal lives, women commonly described working or studying right up until their babies were born. For women who worked in certain industries or jobs, this was a concern for their health, as their positions required strenuous work or had no benefits, but they kept their jobs because of financial needs. In fact, women who participated in the study commonly experienced not having enough money for monthly expenses, including food, which represented a significant barrier to being healthy during pregnancy and postpartum.

*“We run a business that’s not doing very well and we live on a farm. I don’t have any extra help. I’ve got two little kids that I’m trying to raise and, you know, keep food on the table and a clean home for the family (…) I feel overwhelmed every day.” (Pregnant woman, mother of two)*

The stress of working and living with financial difficulties while pregnant was even greater among those who already had children than those having their first child, in part because they could not access affordable childcare in rural areas. Women’s residence in rural communities was also described as placing an additional structural barrier to their health. Prenatal care was not offered in all rural communities, and for some women this meant having to take the day off work, to drive for a few hours and to spend money on gas for frequent medical appointments (especially in their third trimester). For women in our study the apparent simple act of attending prenatal appointments could be immensely complicated by their difficult life circumstances. Moreover, the structural barrier of living in a rural area without adequate access to maternity care was exacerbated when the women experienced pregnancy or birth complications (e.g., preeclampsia, placenta previa, and birth by cesarean section) that forced them to leave their rural communities, and drive to larger centres for prenatal care and birth.

### Relieving the burden of adversity

Notwithstanding the barriers to being healthy during pregnancy and postpartum, women across all rural communities described HMHB supports as facilitators to being healthy. HMHB supports included cooking circles, fresh food boxes (approximately 20 pounds of fruit and vegetables that were subsidized by HMHB), food coupons, and one-on-one time with HMHB providers during home visits or programming. These supports increased women’s opportunities to access, and eat, healthy foods during pregnancy and postpartum. The opportunity to eat healthily created through one of these supports is described below:

*“The fresh food boxes are really helpful because food is just so expensive now; you don’t get the opportunity to buy as much healthy food as you would prefer to buy because you just can’t afford it. So, that’s really helpful, I find.” (Pregnant woman, mother of two)*

Women’s appreciation for the healthy foods offered through HMHB supports was evident in focus groups. Although these food supports did not address the social inequities underlying their lack of sufficient income, they operated as a gateway for social support from HMHB providers. Food supports opened a door into women’s lives, enabling HMHB providers to build meaningful relationships with the women and support them in ways they needed. In the following, a postpartum woman described how a HMHB provider helped her when she felt she was not able to complete what seemed to be a simple task of applying online for employment insurance (EI):

*“[Provider name] came to my house to set up my EI for me because my baby was already two weeks old and I wasn’t doing anything. So she came to my house and got me going on that, I didn’t have to go anywhere.” (Postpartum woman, mother of one)*

We also observed the importance of social support during cooking circles as we embraced the role of observer-as-participant in of them, and heard from a postpartum woman that HMHB providers had become her “family” since her enrollment in the program. In a FG, another participant added how HMHB providers were always available and willing to help her:

*“If there were questions I had on about absolutely anything, I could ask them [HMHB providers]. They were more than willing to help me find the answer and provide me with resources to find it myself which was very helpful in certain cases.” (Postpartum woman, mother of three)*

HMHB providers seemed proud of what was offered to the women in rural communities through cooking circles and fresh food boxes in terms of nutrition and skill building. Nonetheless, our interviews with them elucidated two additional pivotal ways in which HMHB as a community-based program supported rural women in the pre- and post-natal periods: crisis management and navigation to other services and supports.

HMHB providers described how women commonly used the safe space of cooking circles to share their struggles with spousal relationships, parenting, and financial resources. In such instances, the social and emotional support provided through cooking circles became central and could turn into what providers described as crisis management. In crisis circumstances, food cooked together or ordered through fresh food boxes became the entryway for HMHB providers to approach women, schedule home visits, schedule appointments for women with additional social and health services, and explore many issues they were facing in a non-threatening way:

*“I think honestly listening is just one of the biggest things because a lot of them - and I think to try not to judge, really, because everybody has their own story and everybody has their own reasons for where they are and how they came to that. And I just try to let them feel comfortable that that doesn’t matter, that I’m here for them for right now. Their past is of course important and it’s been a part of their life but it’s not what we’re dealing with right now I guess.” (HMHB outreach worker)*

By focusing on women’s needs, providers could support women, and when needed, foster desired changes in their lives. This was mostly accomplished by connecting women to housing services for low income families, local food banks and programs providing women with support beyond 6 months postpartum (when they could no longer access HMHB activities and services), and governmental benefit programs. HMHB providers noted another main role they had was to help women navigate, and access other types of services and supports available for rural communities. All providers identified “referring” clients to other programs or health care providers as part of their work. This was in part possible because HMHB providers had many years of experience and great familiarity with local community stakeholders and social enterprises, as described by the outreach worker: *“I’m kind of the person that has all that knowledge in my head and passes it along.”*

Despite the significance of the navigation to other services and supports, HMHB providers commonly described they did not have enough time to provide health education for each client, which they perceived as a failure in terms of health promotion. Contrary to providers’ perceptions, it was clear through the women’s descriptions of the program and our own observations that providers’ grassroots approach to practice provided much needed supports to women, with the potential of promoting their health in meaningful ways.

HMHB providers offered supports to pregnant and postpartum rural women in a way that was respectful and kept women’s well-being at the centre of the conversation. Overall, thinking of a web of factors shaping rural women’s experiences during pregnancy and postpartum, we can imagine an intricate spider web where women are placed in the centre. While the inner threads represent the many factors shaping women’s lives and experiences, the very outer thread represents women’s perceptions of health (emotional well-being, being physically active, smoking cessation, eating healthily, and taking prenatal vitamins). To get to the outer thread, women must navigate the other inner threads. This is illustrated in Fig. 2 where we captured factors that shaped rural women’s experiences during pregnancy and postpartum.

## Discussion

The perceptions of health among pregnant and postpartum women across rural communities encompassed an overall understanding of health behaviours that contribute to being healthy in pregnancy. In our study, women’s perceptions of a healthy diet reflected key nutrition messages pregnant and postpartum women in Alberta might receive through printed resources or public health programs based on a set of books entitled “Healthy Parents Healthy Children” [23].

Pregnancy has been widely regarded as a “teachable moment” in which women are more likely to engage in healthy behaviours due to increased perceptions of personal risk and outcome expectancies for themselves and their babies, strong affective and emotional responses, and redefined self-concept and social roles [24–27]. However, framing pregnancy as a “teachable moment” places more emphasis on women’s motivation to change lifestyle choices and health behaviours rather than on their experiences in the midst of many difficult life circumstances. As Olander and colleagues [26] propose, we need to look at pregnancy “beyond a teachable moment” and try to better understand how women’s capabilities (e.g., being able to manage nausea and tiredness) and opportunities (e.g., being able to access and afford healthy foods) shape their health behaviours throughout pregnancy and postpartum. For women in our study, pregnancy was perceived as a “cue to action” with many factors acting as barriers to action [27, 28]. These women’s health experiences were a result of what Veenstra and Burnett [29] describe as “codependent dynamic between agency and structure” (p. 210) where women tried to be agents of their own health but coped with structures posed by difficult life circumstances happening parallel to their pregnancy and postpartum. Living in a rural area represented a structural barrier for some women. Studies in North America and Australia have noted that women who live in rural areas face additional challenges in accessing health services during pregnancy and postpartum with great focus on women’s geographic location and transportation issues [5, 15, 30–33]. In our study, the central issue of “rurality” was that it was overlaid by other difficult life circumstances during pregnancy and postpartum, and commonly aggravated women’s limited financial resources because of the need to take time off work and to spend money on transportation to attend prenatal appointments.

The Society of Obstetricians and Gynaecologists of Canada (SOGC) noted in a joint position paper on “Rural Maternity Care” that in instances in which women need to leave their community for birth, they “should be supported both financially and emotionally” [31]. This was not the case among women who participated in our study as there were no additional financial supports to offset the cost and emotional burden of traveling for maternity care [31]. The delivery of HMHB as a CPNP program in rural Alberta, however, enabled providers to facilitate the women’s health in pregnancy and postpartum. Yet, HMHB health care and service providers perceived their lack of time to provide health education for each client as a failure to do preventive health promotion. This perception might be a result of what Raphael [34] describes as society’s limited understanding of the real, practical implications of the social determinants health in someone’s lived experience as we tend to think of people in adverse living conditions as being at a greater risk for engaging in unhealthy lifestyle behaviours. Moreover, HMHB health care and service providers regularly received lifestyle messaging by the provincial health authority and media, which could influence providers’ perception of duty to cover health education in their programming, and in one-on-one time with clients.

Some of the successes of HMHB we found in this study were also described in the 10-year evaluation of CPNP, which emphasized the role of food “in drawing the community together and in creating a safe space” [12]. We observed the drawing together in a safe space during cooking circles, and health care and service providers noted that social and emotional supports were key aspects of the program. With this approach, HMHB had a greater potential in positively affecting women’s lives by nurturing the changes women desired for themselves and their babies. Other programs delivered through community health settings using a group model for prenatal care have also shown various benefits and improved health outcomes for women, including better mental health, satisfaction with care, and parental knowledge [35].

This study followed rigorous principles of qualitative inquiry and provided a thorough description of rural women’s health experiences in light of their difficult life circumstances. However, some limitations must be noted. We had a relatively small sample of 28 women and 8 providers connected to a community-based program in rural Alberta. This might pose a limitation to generalizability of findings; however, our findings can still provide valuable insights to programs and providers working with groups of women facing similar life circumstances in comparable contexts. In addition, we did not collect any demographic information that would allow us to analyze data in relation to women’s income or life circumstances. The limited data on the women’s characteristics were collected through researchers’ observations, which could represent a challenge to the validity of these data.

## Conclusions

Women’s perceptions of health, and examples of how they tried to achieve such, showed they wanted to do best for their and baby’s health but faced numerous difficult life circumstances during pregnancy and postpartum. Despite existing challenges, programs, such as HMHB, can play a critical role in helping women to mediate some of these difficult circumstances. Women who participated in the program received much needed additional health and social support from providers who understood their life contexts in a non-judgemental way. When community-based programs show such potential to alleviate some of women’s burdens in coping with difficult life circumstances, they should be well supported through policies and expanded to other locations to increase reach. Indeed, policies that support community-based programs in rural communities, and ensure adequate funding, can enable more equitable services to rural women, and truly promote maternal health during pregnancy and postpartum.

## Additional file

Additional file 1: Women’s Focus Group Guide. This file includes questions the authors explored with women who participated in focus group discussions. It is worth noting that questions were not asked in order shown and as written because participants naturally approached subject areas as they exchanged ideas and experiences during focus groups. (DOCX 21 kb)

## Abbreviations

CBPR: Community-Based Participatory Research
CPNP: Canada Prenatal Nutrition Program
EI: Employment Insurance
FGs: Focus Groups
HMHB: Healthy Moms Healthy Babies
PHAC: Public Health Agency of Canada
SOGC: Society of Obstetricians and Gynaecologists of Canada

## References

[1] Nash DM, Gilliland JA, Evers SE, Wilk P, Campbell MK (2013). Determinants of diet quality in pregnancy: sociodemographic, pregnancy-specific, and food environment influences. J Nutr Educ Behav.

[2] Barker D, Barker M, Fleming T, Lampl M (2013). Developmental biology: support mothers to secure future public health. Nature.

[3] Paul KH, Graham ML, Olson CM (2013). The web of risk factors for excessive gestational weight gain in low income women. Matern Child Health J.

[4] Urquia ML, Glazier RH, Blondel B, Zeitlin J, Gissler M, Macfarlane A, Ng E, Heaman M, Stray-Pedersen B, Gagnon AJ (2010). International migration and adverse birth outcomes: role of ethnicity, region of origin and destination. J Epidemiol Community Health.

[5] Sutherns R, Bourgeault IL (2008). Accessing maternity care in rural Canada: there’s more to the story than distance to a doctor. Health Care Women Int.

[6] Tarasuk V, Mitchell A, Dachner N. Household food insecurity in Canada, 2014. Research to identify policy options to reduce food insecurity (PROOF). 2014. Available from: http://nutritionalsciences.lamp.utoronto.ca/. Accessed 16 Feb 2017.

[7] Hromi-Fiedler A, Bermudez-Millan A, Segura-Perez S, Perez-Escamilla R (2011). Household food insecurity is associated with depressive symptoms among low-income pregnant Latinas. Matern Child Nutr.

[8] Laraia BA, Siega-Riz AM, Gundersen C, Dole N (2006). Psychosocial factors and socioeconomic indicators are associated with household food insecurity among pregnant women. J Nutr.

[9] Laraia B, Epel E, Siega-Riz AM (2013). Food insecurity with past experience of restrained eating is a recipe for increased gestational weight gain. Appetite.

[10] Laraia BA, Siega-Riz AM, Gundersen C (2010). Household food insecurity is associated with self-reported pregravid weight status, gestational weight gain, and pregnancy complications. J Am Diet Assoc.

[11] Public Health Agency of Canada. Canadian Prenatal Nutrition Program (CPNP) 2015. Available from: http://www.phac-aspc.gc.ca/hp-ps/dca-dea/prog-ini/cpnp-pcnp/about-apropos-eng.php. Accessed 18 Jun 2017.

[12] Public Health Agency of Canada (2007). The Canada Prenatal Nutrition Program: A decade of promoting the health of mothers, babies and communities.

[13] Public Health Agency of Canada. CPNP Projects Directory Online 2011. Available from: http://cpnp-pcnp.phac-aspc.gc.ca/index-eng.php. Accessed 18 Jun 2017.

[14] Quintanilha M, Mayan MJ, Thompson J, Bell RC (2016). Contrasting “back home” and “here”: how northeast African migrant women perceive and experience health during pregnancy and postpartum in Canada. Int J Equity Health.

[15] Sutherns R (2005). So close yet so far: rurality as a determinant of women’s health. Can Woman Stud.

[16] Minkler M, Wallerstein N (2008). Community-based participatory research for health: from process to outcomes.

[17] Knoblauch H. Focused Ethnography. Forum Qual Soc Res. 2005;6(3):1-14.

[18] Higginbottom GMA, Pillay JJ, Boadu NY (2013). Guidance on performing focused ethnographies with an emphasis on healthcare research. Qual Rep.

[19] Patton MQ (2002). Qualitative research & evaluation methods.

[20] Hennink MM (2007). International focus group research: a handbook for the health and social sciences.

[21] Mayan MJ (2009). Essentials of qualitative inquiry.

[22] Hsiu-Fang H, Shannon SE (2005). Three approached to qualitative content analysis. Qual Health Res.

[23] Alberta Health Services. Healthy Parents Healthy Children: Pregnancy and Birth. Available from: http://www.healthyparentshealthychildren.ca/. Accessed 24 Nov 2017.

[24] Atkinson L, Shaw RL, French DP (2016). Is pregnancy a teachable moment for diet and physical activity behaviour change? An interpretative phenomenological analysis of the experiences of women during their first pregnancy. Br J Health Psychol.

[25] Phelan S (2010). Pregnancy: a “teachable moment” for weight control and obesity prevention. Am J Obstet Gynecol.

[26] Olander EK, Darwin ZJ, Atkinson L, Smith DM, Gardner B (2016). Beyond the ‘teachable moment’ - a conceptual analysis of women’s perinatal behaviour change. Women Birth.

[27] Lawson PJ, Flocke SA (2009). Teachable moments for health behavior change: a concept analysis. Patient Educ Couns.

[28] Geller PA (2004). Pregnancy as a stressful life event. CNS Spectr.

[29] Veenstra G, Burnett PJ (2016). Towards a relational health promotion. Health Promot Int.

[30] Goodwin JW (1999). The great Canadian rural obstetrician meltdown. J Soc Obstet Gynaecol Can.

[31] Miller KJ, Couchie C, Ehman W, Graves L, Grzybowski S, Medves J (2012). Rural maternity care. J Obstet Gynaecol Can.

[32] Hoang H, Le Q, Ogden K (2014). Women’s maternity care needs and related service models in rural areas: a comprehensive systematic review of qualitative evidence. Women Birth.

[33] Gjesfjeld CD, Jung JK (2011). How far?: using geographical information systems (GIS) to examine maternity care access for expectant mothers in a rural state. Soc Work Health Care.

[34] Raphael D, Raphael D (2016). Social determinants of health: key issues and themes. Social determinants of health: Canadian perspectives.

[35] Kania-Richmond A, Hetherington E, McNeil D, Bayrampour H, Tough S, Metcalfe A (2017). The impact of introducing centering pregnancy in a community health setting: a qualitative study of experiences and perspectives of health center clinical and support staff. Matern Child Health J.

