# Supplementary material for: Nurturing maternal health in the midst of difficult life circumstances: a qualitative study of women and providers connected to a community-based perinatal program
Source: BMC Pregnancy Childbirth. 2018 Aug 3;18:314. doi: 10.1186/s12884-018-1951-6 (PMC6091112; doi:10.1186/s12884-018-1951-6)
Supplement: Supplementary file 1 — Women’s Focus Group Guide. This file includes questions the authors explored with women who participated in focus group discussions. It is worth noting that questions were not asked in order shown and as written because participants naturally approached subject areas as they exchanged ideas and experiences during focus groups. (DOCX 21 kb) [file 12884_2018_1951_MOESM1_ESM.docx]

**Women’s Focus Group Guide**

Thank you for all taking the time to talk to me today. Today I want to learn about your feelings and thoughts regarding your health during and after pregnancy, and how to better support you in these areas.

I would like to remind everyone that what is shared today is confidential and to please respect others in the group by keeping comments private. If anyone does not feel comfortable answering any questions within the group, you do not have to.

**Pregnancy & Postpartum**

1. What does it mean to you to be healthy during pregnancy? [Probe for social norms, cultural beliefs, and community/family support]
   1. How about after the baby is born?
2. What drives/shapes your health decisions in pregnancy and postpartum? [Explore challenges and supports to health decisions, **including those related to nutrition**]
3. What types of foods do you eat during pregnancy? [Probes: cravings, food aversions, specific nutrients]
4. How were your visits to health care providers’ office (e.g., doctors, nurses, midwives, etc.) during and after pregnancy? [Probes: needs, expectations]
   1. How often did you see this health care provider?
   2. What information about health and nutrition did your health care provider give you during the visits?
5. Community-based organization context: what was your experience with this program (HMHB) during pregnancy and postpartum?
6. If anything, what would you need to be/feel healthier during pregnancy and after you give birth?
7. What do you know now that you wish you knew before your first pregnancy?
